# Sociodemographic Determinants of Urogenital Morbidities Among Menopausal Women in Rural Areas of Eastern Uttar Pradesh

**DOI:** 10.7759/cureus.46677

**Published:** 2023-10-08

**Authors:** Shalini Singh, Kavita Baranwal, Indu Rana, Imran Ahmed Khan, Somesh Bajpai

**Affiliations:** 1 Community Medicine, Baba Raghav Das Medical College, Gorakhpur, IND

**Keywords:** urinary symptoms, postmenopausal, morbidity, menopausal transition, genital symptoms

## Abstract

Background

After their mid-forties, almost all women, irrespective of their cultural background and health conditions, begin to experience physical, psychological, and emotional disturbances because of the progressive decline in hormone levels that occur as a reproductive-aged woman transitions from regular cyclic menses to her final menstrual period, ovarian senescence, and beyond. These morbidities hamper day-to-day life and lead to poor quality of life. Timely attention and management of these morbidities help women maintain a healthy and active life. This study aims to evaluate sociodemographic determinants of urogenital morbidities among rural menopausal women.

Materials and methods

We conducted the present cross-sectional study among a menopausal transition group and a postmenopausal group of women age 40 to 55 residing in the Chargawan block of the district of Gorakhpur from August 1, 2021, to July 31, 2022. After estimating the sample size based on the 2011 census of India, we selected 385 eligible participants.

Results

We studied a total of 385 women over a period of one year, out of which 171 (46%) were in the menopausal transition and 214 (54%) were postmenopausal. For urinary incontinence and burning micturition, when we compared both groups in relation to age, we found no significant association (p > .05). The symptom of urinary incontinence was significantly associated with the socioeconomic status of participants in both the menopausal transition and postmenopausal groups (p < .05).

Conclusions

Postmenopausal women harbor a considerable number of urogenital morbidities. Sociocultural, demographic, and behavioral factors influence these morbidities. These associations might serve as indicators of women at risk of experiencing more severe urogenital morbidities.

## Introduction

Hormones play a vital role in body homeostasis. Different hormones are responsible for this harmony. Researchers have identified and studied various hormones in detail, including growth hormone, thyroid hormone, parathyroid hormone, luteinizing hormone, estrogen, progesterone, and insulin. Though produced in very minute quantities, these hormones are essential to life. Along the same lines, female hormones play a central role in women’s lives. Their rise triggers puberty, allowing women to experience the joy of motherhood and ensure cardioprotective functions and bone health [1,2].

Increasing age produces various changes in this balance. Subsequently, a progressive decline in hormone levels occurs as a reproductive-aged woman transitions from regular cyclic menses to her final menstrual period, ovarian senescence, and beyond [3]. After their mid-forties, almost all women, irrespective of their cultural background and health conditions, begin to experience physical, psychological, and emotional disturbances because of a progressive endocrinologic decline in hormone levels [4,5]. These changes also affect pelvic floor anatomy and function, resulting in several disorders, such as weakness and prolapse of pelvic organs, bowel and bladder dysfunction, recurrent lower urinary tract symptoms, and sexual dysfunction [6]. These morbidities hamper day-to-day life and lead to poor quality of life [7]. Timely attention and management of these morbidities help women maintain a healthy and active life. This study aims to evaluate sociodemographic determinants of urogenital morbidities among rural menopausal women in eastern Uttar Pradesh.

## Materials and methods

Study design

A cross-sectional study approved by the research council of BRD Medical College, Gorakhpur (Reference No. 58/CRC/2021) was conducted among women aged 40 to 55 in the menopausal transition or postmenopausal phase of their lives residing in the rural areas of the Chargawan block from August 1, 2021, to July 31, 2022.

Study definitions

Menopause denotes the cessation of menses for a period of 12 months or more in a woman aged 40 or older. The menopause transition includes irregular menstrual cycles in which the interval between cycles may be altered by seven or more days, two or more skipped cycles, and at least one intermenstrual interval of 60 days or more. [5] Vegetarians were those who had a vegetarian diet only. Occasional nonvegetarians were having a nonvegetarian diet weekly, fortnightly, or monthly, whereas regular nonvegetarians were having a nonvegetarian diet at least twice per week. The “yes” category of exercise included participants who walked for at least 30 minutes five days per week. The socioeconomic classification was according to the modified B. G. Prasad classification, using the All India Consumer Price Index of May 2021 as 119.6.

Inclusion and exclusion criteria

Menopausal women aged 40 to 55 who are available in that specific area and willing to participate in the study

Menopausal women who were not willing to give consent to participate in the study-those having a major systemic illness; menopausal women with a known history of surgical hysterectomy or any kind of hormonal therapy; and patients who were unable to respond to questions due to a high physical or mental disability limiting their participation in the study.

Data collection

We conducted this cross-sectional study among a menopausal transition group and a postmenopausal group of women aged 40 to 55 residing in the Chargawan block of the district of Gorakhpur. We estimated the sample size based on the 2011 census of India, in which the prevalence of vasomotor symptoms among menopausal women was 60.7% [8]. Considering the prevalence of vasomotor symptoms at 60.7%, an allowable error of 5%, and a 95% confidence interval, we calculated the sample size using Cochran’s formula and obtained a sample size of 367. Considering 10% of the nonresponse rate, a total of 403 participants were taken. Chargawan Block has one block-level PHC and four additional PHCs. We selected Chargawan PHC randomly. Out of its seven subcenters, we randomly selected two subcenters-Narayanpur and Harsewakpur No. 2-using the lottery method. Then, we randomly selected two villages from each subcenter using the lottery method. Finally, we chose the four villages of Karmahatola, Umarpurtola, Chauhan Tola, and Musalmantola.

In each village, we made house-to-house visits to compile a list of all menopausal women aged 40 to 55 with their names and addresses. The list consisted of 403 participants as a sample population, including 78 from Karmaha, 153 from Umarpurtola, 65 from Chauhan Tola, and 107 from Musalmantola. Out of these 403 women, 14 had a history of hysterectomy; therefore, we excluded them from the list. We informed the rest of the participants (389) about the purpose of the study and obtained the participants’ informed written consent in the local language (Hindi). Out of these 389 participants, four were not willing to give consent, so we excluded them from the study, resulting in a total of 385 eligible participants. These included 171 women in the menopausal transition and 214 women in the postmenopausal group.

With the help of a predesigned and pretested questionnaire, we interviewed each participant in the sample population. We used a digital machine to measure blood pressure and a stadiometer to measure height, with an accuracy of up to 0.5 cm. We also used an electronic machine to measure weight with 0.1 kg accuracy. We entered the data we obtained into the Microsoft Office Excel sheet and coded it. We then imported the data into IBM Corp. Released 2012. IBM SPSS Statistics for Windows, Version 21.0. Armonk, NY: IBM Corp. for further analysis.

Statistical analysis 

We presented the data as mean ± standard deviation (SD) for continuous variables and as percentages (%) for categorical variables. We tested sociodemographic determinants (participants’ age, education level, education level of husbands, occupation, socioeconomic status, and type of family) as independent variables for their effect on urogenital morbidities. We used the Chi-Square test and the Fisher-Exact test (whichever was applicable) as tests of significance.

## Results

Over a period of one year, we studied a total of 385 women, out of whom 171 (46%) were in the menopausal transition and 214 (54%) were in the postmenopausal group. 

Table 1 shows the sociodemographic characteristics of the study participants. The mean age was 43.94 ± 4.74 in the menopausal transition group and 48.89 ± 3.48 in the postmenopausal group. Most participants in both groups belonged to the Hindu religion and were illiterate. Among both groups, most of the women were married. The menopausal transition group included only one unmarried woman. Most women in the menopausal transition group (81, or 47.4%) and postmenopausal group (112, or 52.3%) were from the lower socioeconomic class.

**Table 1:** Sociodemographic profile of participants (n = 385) MT: Menopausal transition; PM: Post-menopausal; SD: Standard deviation; NA: Not applicable

Sociodemographic Factors		MT (n = 171)	PM (n = 214)	
		Number (%)		
Age	40–43	81 (47.4)		26 (12.1)
	44–47	62 (36.3)		64 (29.9)
	48–51	22 (12.9)		48 (22.4)
	52–55	6 (3.5)		76 (35.5)
	Mean ± SD	43.94 ± 3.48		48.89 ± 4.74
Religion	Hindu	157 (91.8)		189 (88.3)
	Muslim	14 (8.2)		25 (11.7)
Caste	General	10 (5.8)		6 (2.8)
	OBC	83 (48.5)		84 (39.3)
	SC/ST	78 (45.6)		124 (57.9)
Education of Participants	Illiterate	120 (70.2)		168 (78.5)
	Primary school	13 (7.6)		19 (8.9)
	Middle school	18 (10.5)		21 (9.8)
	High school	3 (1.8)		5 (2.3)
	Intermediate	9 (5.3)		0
	Graduate/Postgraduate	8 (4.7)		1 (0.5)
Occupation of Participants	Housewife	149 (87.1)		158 (73.8)
	Skilled	4 (2.3)		12 (5.6)
	Semiskilled	9 (5.3)		7 (3.3)
	Unskilled	6 (3.5)		35 (16.4)
	Others	3 (1.8)		2 (0.9)
Marital Status	Unmarried	1 (0.6)		0
	Married	159 (93)		189 (88.3)
	Widowed	11 (6.4)		25 (11.7)
Type of Family	Nuclear	116 (67.8)		113 (52.8)
	Joint	55 (32.2)		101 (47.2)
Socio-economic Status (*Indian Rupee/Capita/Month)	Upper class (I) (≥ 7,863)^ *^	1 (0.6)		2 (0.9)
	Upper middle class (II) (3,931–7,862)	5 (2.9)		9 (4.2)
	Middle class (III) (2,359–3,930)	16 (9.4)		7 (3.3)
	Lower middle class (IV) (1,179–2,358)	68 (39.8)		84 (39.3)
	Lower class (V) (≤1,179)	81 (47.4)		112 (52.3)
Mean age of Menopause	Mean ± SD	NA		46.31 ± 1.86.

Most of the women in the menopausal transition group and postmenopausal group attained menarche at the age of 12 to 14. Ninety-four women (55%) in the transition group and 151 women (70.6%) in the postmenopausal group had more than five children. One hundred twenty-one women (70.8%) in the transition group and 154 women (72%) in the postmenopausal group were not addicted to any kind of agent. Table 2 shows other personal characteristics.

**Table 2:** Distribution of participants according to their personal history and anthropometric measurements (BMI) MT: Menopausal transition; PM: Post-menopausal; SD: Standard deviation; BMI: Body mass index

Participants Characteristics		MT (n = 171)	PM (n = 214)
		Number (%)	
Age of Menarche	12–14	99 (57.9)	138 (64.5)
	15–17	72 (42.1)	76 (35.5)
	Mean ± SD	14.18 ± 1.34	13.87 ± 1.32
Parity	0	4 (2.3)	3 (1.4)
	1–2	31 (18.1)	16 (7.5)
	3–4	42 (24.6)	44 (20.6)
	> 5	94 (55.0)	151 (70.6)
Addiction	Yes	50 (29.2)	60 (28.0)
	No	121 (70.8)	154 (72.0)
Dietary Habits	Occasional nonvegetarian	96 (56.1)	136 (63.6)
	Regular nonvegetarian	56 (32.7)	53 (24.8)
	Vegetarian	19 (11.1)	25 (11.7)
BMI (Kg/m^2^)	< 18.5	13 (7.6)	15 (7.0)
	18.50–24.99	66 (38.6)	92 (43.0)
	25–29.99	47 (27.5)	86 (40.2)
	> 30	45 (26.3)	21 (9.8)
	Mean ± SD	26.49 ± 5.47	25.18 ± 4.58
Exercise	Yes	30 (17.5)	76 (17.7)
	No	141 (82.5)	138 (83.3)

Table 3 depicts the association of urinary symptoms with the sociodemographic profile of the menopausal transition and postmenopausal groups. The participants identified two types of urinary symptoms: urinary incontinence and burning micturition. A comparison of both groups revealed no significant association between urinary symptoms and age (p > .05). In relation to the education of participants, we discovered a significant difference between the groups only for the symptom of burning micturition (p < .01). No significant difference between the groups existed for urinary symptoms in relation to the participants’ occupation. In relation to the husband’s education, we found a highly significant association with the symptom of burning micturition among both groups (p < .001). However, we found a significant association with sociodemographic class only for the symptom of urinary incontinence (p < .05).

**Table 3:** Association of urinary symptoms in relation to the sociodemographic profile of participants MT: Menopausal transition; PM: Post-menopausal; p: Test of significance

Variables	Urinary Incontinence			Burning Micturition		
	MT (n = 21)	PM (n = 29)		MT (n = 25)	PM (n = 36)	
Age Group						
40–43	13 (61.9)	5 (17.2)	p = .275	11 (44.0)	5 (13.9)	p = .988
44–47	5 (23.8)	7 (24.2)		9 (36.0)	12 (33.3)	
48–51	2 (9.5)	5 (17.2)		3 (12.0)	8 (22.2)	
52–55	1 (4.8)	12(41.4)		2 (8.0)	11 (30.6)	
Education of Participants						
Literate	9 (42.9)	2 (6.9)	p = .607	5 (20.0)	6.5 (25.0)	p = .001
Illiterate	12 (57.1)	27 (93.1)		20 (80.0)	19.5 (75.0)	
Occupation of Participants						
Employed	3 (14.3)	4 (13.8)	p = .337	5 (20.0)	6 (16.7)	p = .840
Unemployed	18 (85.7)	25 (86.2)		20 (80.0)	30 (83.3)	
Education of Husband						
Literate	15 (71.4)	14 (48.3)	p = .202	18 (72.0)	9 (25.0)	p < .001
Illiterate	6 (28.6)	15 (51.7)		7 (28.0)	27 (75.0)	
Type of Family						
Nuclear	16 (76.2)	143 (44.8)	p = .819	17 (68.0)	15 (41.7)	p=.223
Joint	5 (23.8)	16 (55.2)		8 (32.0)	21 (58.3)	
Socio-economic Status						
Upper class (Ⅰ)	1 (4.8)	1 (3.4)	p = .007	1 (4.0)	1 (2.8)	p = .220
Upper middle class(Ⅱ)	1 (4.8)	1 (3.4)		1 (4.0)	5 (13.9)	
Middle class (Ⅲ)	5 (23.8)	2 (6.9)		2 (8.0)	1 (2.8)	
Lower middle class(Ⅳ)	6 (28.6)	6 (20.8)		8 (32.0)	14 (38.8)	
Lower class(Ⅴ)	8 (38.0)	19 (65.5)1		13 (52.0)	15 (41.7)	

Table 4 depicts the association between loss of libido and vaginal dryness in relation to the sociodemographic profile of the participants. In the youngest age range (40-43) of the menopausal transition group, 10 participants (47.6%) identified loss of libido and 12 participants (70.6%) identified vaginal dryness as symptoms. In the postmenopausal group, both of these symptoms were maximal in the eldest age range (52-55), with 20 participants (31.3%) identifying loss of libido and 21 participants (33.3%) identifying vaginal dryness as symptoms. However, we found no significant difference between the two groups. A statistically significant difference (p <.05) existed between the two groups in the association of vaginal dryness with socioeconomic class.

**Table 4:** Association of genital symptoms in relation to the sociodemographic profile of the participants MT: Menopausal transition; PM: Post-menopausal; p: Test of significance

Variables	Loss of Libido						Vaginal Dryness		
	MT (n = 21)				PM (n = 64)		MT (n = 17)	PM (n = 63)	
Age Group (Years)									
40–43	10 (47.6)			11 (17.2)		p = .181	12 (70.6)	10 (15.9)	p = .450
44–47	9 (42.8)			13 (20.2)			3 (17.6)	18 (28.6)	
48–51	1 (4.8)			20 (31.3)			1 (5.9)	14 (22.2)	
52–55	1 (4.8)			20 (31.3))			1 (5.9)	21 (33.3)	
Education of Participants									
Literate	10 (47.6)	8 (12.5)				p = .364	8 (47.1)	11 (17.5)	p = .783
Illiterate	11 (52.4)	56 (87.5)					9 (52.9)	52 (82.5)	
Occupation of Participants									p = .783
Employed	1 (4.8)	5 (7.8)				p = .002	8 (47.1)	11 (17.5)	
Unemployed	20 (95.2)	59 (92.2)					9 (52.9)	52 (82.5)	
Education of Husband									
Literate	16 (76.2)	34 (53.1)				p = .78	14 (82.4)	31 (49.2)	p = .039
Illiterate	5 (23.8)	30 (46.9)					3 (17.6)	32 (50.8)	
Type of Family									
Nuclear	19 (90.5)	35 (54.7)				p = .390	16 (94.1)	34 (54.0)	p = .537
Joint	2 (9.5)	29 (45.3)					1 (5.9)	29 (46.0)	
Socioeconomic Status									
Upper class (Ⅰ)	1 (4.8)		2 (3.1)			p = .009	1 (5.9)	1 (1.6)	p = .016
Upper middle class (Ⅱ)	1 (4.8)		5 (7.8)				1 (5.9)	6 (9.5)	
Middle class(Ⅲ)	5 (23.8)		2 (3.1)				5 (29.4)	1 (1.6)	
Lower middle class (ⅠV)	9 (42.8)		25 (39.1)				5 (29.4)	29 (46.0)	
Lower class (Ⅴ)	5 (23.8)		30 (46.9)				5 (29.4)	26 (41.3)	

## Discussion

The aim of our study was to identify factors that contribute to urogenital morbidities at the menopausal stage of life. The mean age at which participants attained menopause was 46.31 ± 1.86, which was almost similar to the finding of Indian menopausal society, i.e., 46 [9]. The relationship between sociodemographic factors such as age, education, occupation, type of family, income, and urogenital morbidities is unclear and debated.

This study found urinary incontinence in 21 women (12.3%) in the menopausal transition group and 29 postmenopausal women (13.6%). A similar study by Mathew et al. [10] reported that 18.7% had urinary incontinence. In another study by Goyal A. et al. [11], 9.0% of rural females and 7.5% of urban females reported increased urinary frequency, which is comparatively less than our findings. In a study by Karmakar et al. [12], 63% of females reported increased urinary frequency. 

In our study, 17 women (9.9%) in the menopausal transition group and 63 women (29.4%) in the postmenopausal group reported vaginal dryness. Loss of libido was a symptom for 21 women (12.3%) in the menopausal transition group and 64 women (29.9%) in the postmenopausal group. Mathew et al. [10] reported that 59 women (18.7%) had complaints of vaginal dryness, which was almost similar to this study. In another study, Singh and Pradhan [13] observed decreased libido in 85 women (33.7%) and vaginal dryness in 51 women (20.2%), which is higher than our study. 

Sangeeta Ramteke et al. [14] found that sexual problems were most common among women of postmenopausal age, i.e., 86% of women, in the form of loss of interest in sexual activity, pain during intercourse, and loss of libido. Most postmenopausal women experience a decrease in sexual drive because a lack of estrogen causes atrophy of the vagina and emotional changes. Menopausal women can have urinary incontinence as a morbidity, but they are unaware that these morbidities can be managed. 

Participants’ education had a substantial impact on the occurrence of symptoms of burning micturition. The lower educational levels of participants and their husbands may be contributing factors to the severity of morbidities. Employment was a statistically significant determinant only for the symptom of loss of libido (p <.01). Urogenital symptoms were visibly less common among employed women. Women’s stress and anxiety in the workplace, along with their hormonal changes, may contribute to dissatisfaction. Even though some aspects of employment can affect sexual life, ceasing to work usually has negative effects on health. Women from lower socioeconomic strata are limited in their ability to deal with morbidities effectively because of limited access to resources, and they do not report their condition or seek treatment. This may be due to the initial withdrawal of hormones, which makes the menopausal transition group more sensitive to these changes in comparison to the postmenopausal group because they adapt to these changes and overcompensate for the symptoms. A higher level of education, higher socioeconomic status, and social support provide better prospects for having a positive attitude toward this period of life. 

Our study has a few limitations. Some participants may not declare their whole spectrum of morbidities due to cultural constraints. Because this is a cross-sectional study, we cannot establish a causal relationship. Another limitation may be recall bias because the participants may have underreported or overreported information.

## Conclusions

Most of the women in the menopausal transition group and postmenopausal group attained menarche at the age of 12 to 14. Postmenopausal women harbor a considerable number of urogenital morbidities. Urinary incontinence and burning micturition were the predominant urinary symptoms. The symptom of urinary incontinence was significantly associated with the socioeconomic status of participants in both the menopausal transition and postmenopausal groups (p <.05). Common urogenital symptoms were loss of libido and vaginal dryness. The sociodemographic factors predisposing to urogenital morbidities in menopausal women include lower education, unemployment, and lower socioeconomic status. Being unaware of symptoms and having limited access to resources are barriers to achieving positive health. These associations might serve as indicators of women at risk of experiencing more severe urogenital morbidities and guide some appropriate management strategies.
